# Preliminary data set to assess the performance of an outdoor membrane photobioreactor

**DOI:** 10.1016/j.dib.2019.104599

**Published:** 2019-10-04

**Authors:** J. González-Camejo, A. Jiménez-Benítez, M.V. Ruano, A. Robles, R. Barat, J. Ferrer

**Affiliations:** a Calagua – Unidad Mixta UV-UPV, Institut Universitari d'Investigació d’Enginyeria de l'Aigua i Medi Ambient – IIAMA, Universitat Politècnica de València, Camí de Vera s/n, 46022 Valencia, Spain; b Calagua – Unidad Mixta UV-UPV, Departament d'Enginyeria Química, Universitat de València, Avinguda de la Universitat s/n, 46100 Burjassot, Valencia, Spain

**Keywords:** Ammonium limitation, Growth rate, Membrane photobioreactor, Outdoor, Turbidity

## Abstract

This data in brief (DIB) article is related to a Research article entitled ‘Optimising an outdoor membrane photobioreactor for tertiary sewage treatment’ [1].

Data related to the effect of substrate turbidity, the ammonium concentration at which the culture reaches nitrogen-deplete conditions and the microalgae growth rate under outdoor conditions is provided.

Microalgae growth rates under different substrate turbidity were obtained to assess the reduction of the culture's light availability. Lab-scale experiments showed growth rates reductions of 22–44%.

Respirometric tests were carried to know the limiting ammonium concentration in this microalgae-based wastewater treatment system.

Growth rates (μ) of green microalgae *Scenedesmus* and *Chlorella* obtained under outdoor conditions; i.e. 0.40 d^−1^ (R^2^ = 0.993) and 0.43 d^−1^ (R^2^ = 0.995), respectively, can be useful to obtain optimum operating conditions of membrane photobioreactor (MPBR).

**Table:** Specifications Table

Subject area	*Water Science and Technology*
More specific subject area	*Microalgae-based wastewater treatment*
Type of data	*Table* *Image* *Graph*
How data was acquired	*Microalgae growth was obtained by the slope of the microalgae biomass evolution. Oxygen production rates were obtained by respirometric tests. Growth rates under outdoor conditions were calculated by the evolution of the optical density of* 680nm.
Data format	*Raw* *Analysed*
Parameters for data collection	*Grab samples were collected every certain amount of time. This time varied according to the experiment.*
Description of data collection	*Grab samples were collected from the microalgae culture. The culture was considered to be adequately mixed.*
Data source location	*Alboraya, Valencia* *Spain* *39°30′04.0″N 0°20′00.1″W*
Data accessibility	*With this article*
Related research article	*González-Camejo, J., Jiménez-Benítez, A., Ruano, M.V., Robles, A., Barat, R., Ferrer, F., 2019. Optimising an outdoor membrane photobioreactor for tertiary sewage treatment. J. Environ. Manag. 245, 76–85.*https://doi.org/10.1016/j.jenvman.2019.05.010

**Table:** 

**Value of the Data** • The effect of high and low turbidity in the substrate, which is related to the culture's light availability, can be evaluated. • The data enables to assess the ammonium concentration which limits microalgae activity. • Growth rates could be used to compare different microalgae species and cultivation systems. • Growth rates can be used to obtain the theoretically optimal biomass retention time (BRT) and hydraulic retention time (HRT) [2]. • This data highlights some relevant aspects that influence the operation of a microalgae cultivation system. • Data of this DiB article can be applied to outdoor microalgae cultivation systems.

## 1 Data

Several tests were elaborated: i) substrate turbidity; ii) the ammonium concentration at which the culture reaches nitrogen-deplete conditions; and iii) the microalgae growth rate under outdoor conditions.

**Fig. 1:**
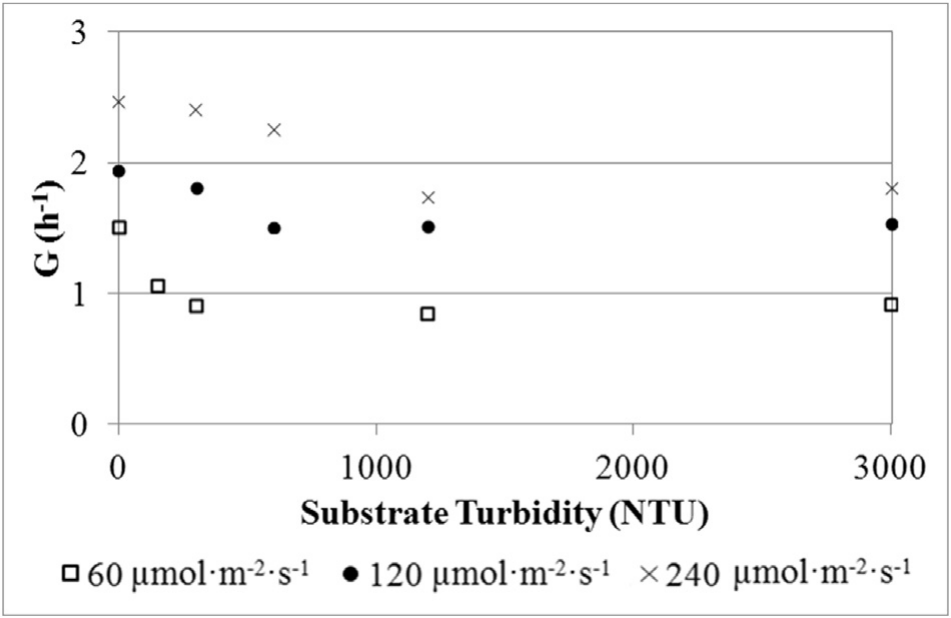
Microalgae growth at different light intensities.

To see the substrate turbidity effect on microalgae, culture growth rate was measured at different turbidity values (Fig. 1). Raw data regarding the evolution of optical density which was used to calculate microalgae growth can be found in Supplementary material.

**Fig. 2:**
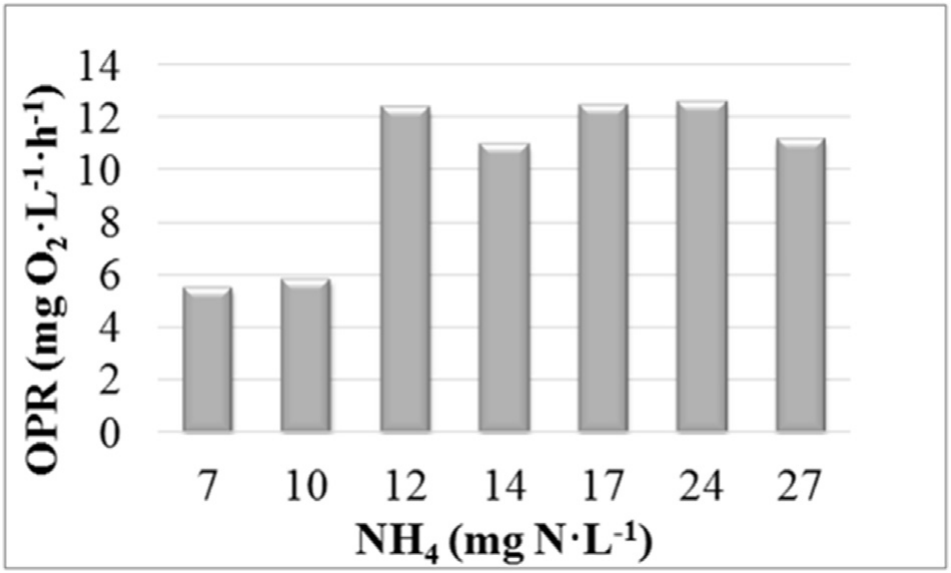
Oxygen production rates (OPRs) obtained during the respirometric tests at different ammonium (NH_4_) concentrations.

Respirometric tests were carried out under different nitrogen concentrations to obtain the oxygen production rates (OPR) (Fig. 2). Raw data regarding the evolution of oxygen (which was used to calculate OPRs) can be found in Supplementary material.

**Fig. 3:**
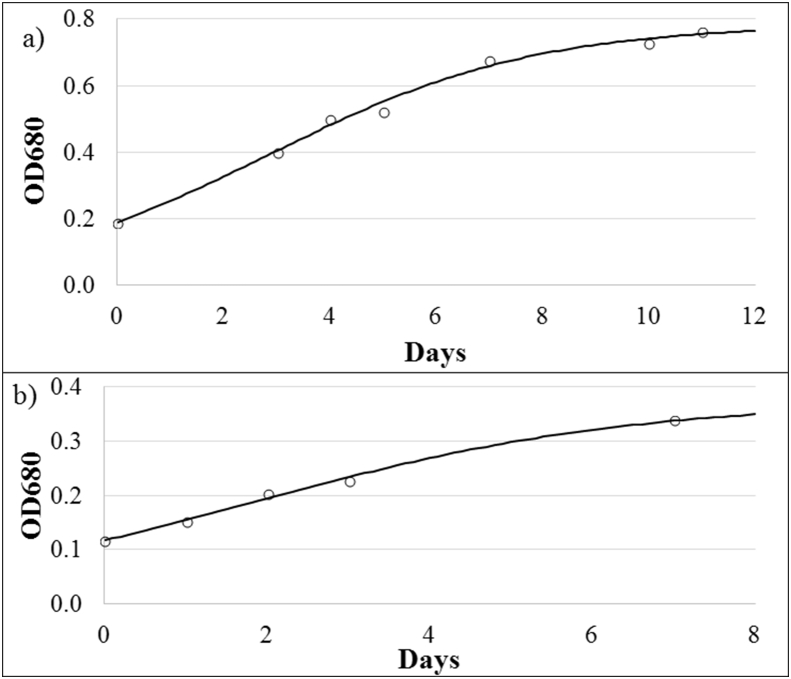
Evolution of optical density at 680 nm (OD680) during: a) Assay a (batch stage of Experiment BRT4.5 [1]) (*Scenedesmus*-dominated) and b) Assay b (batch stage of Experiment HRT3.5 [1]) (*Chlorella*-dominated).

The growth rate of microalgae cultures can be very helpful to operate microalgae cultivation systems, since maximum biomass productivity is reached when biomass retention time (BRT) equals 2·μ^−1^ [2]. Growth rates can be obtained by the time-evolution of the culture's optical density (see Supplementary Material). Growth rates of 0.40 d^−1^ (R^2^ = 0.993) and 0.43 d^−1^ (R^2^ = 0.995), were observed for Assay a (Fig. 3a) and b (Fig. 3b).

**Fig. 4:**
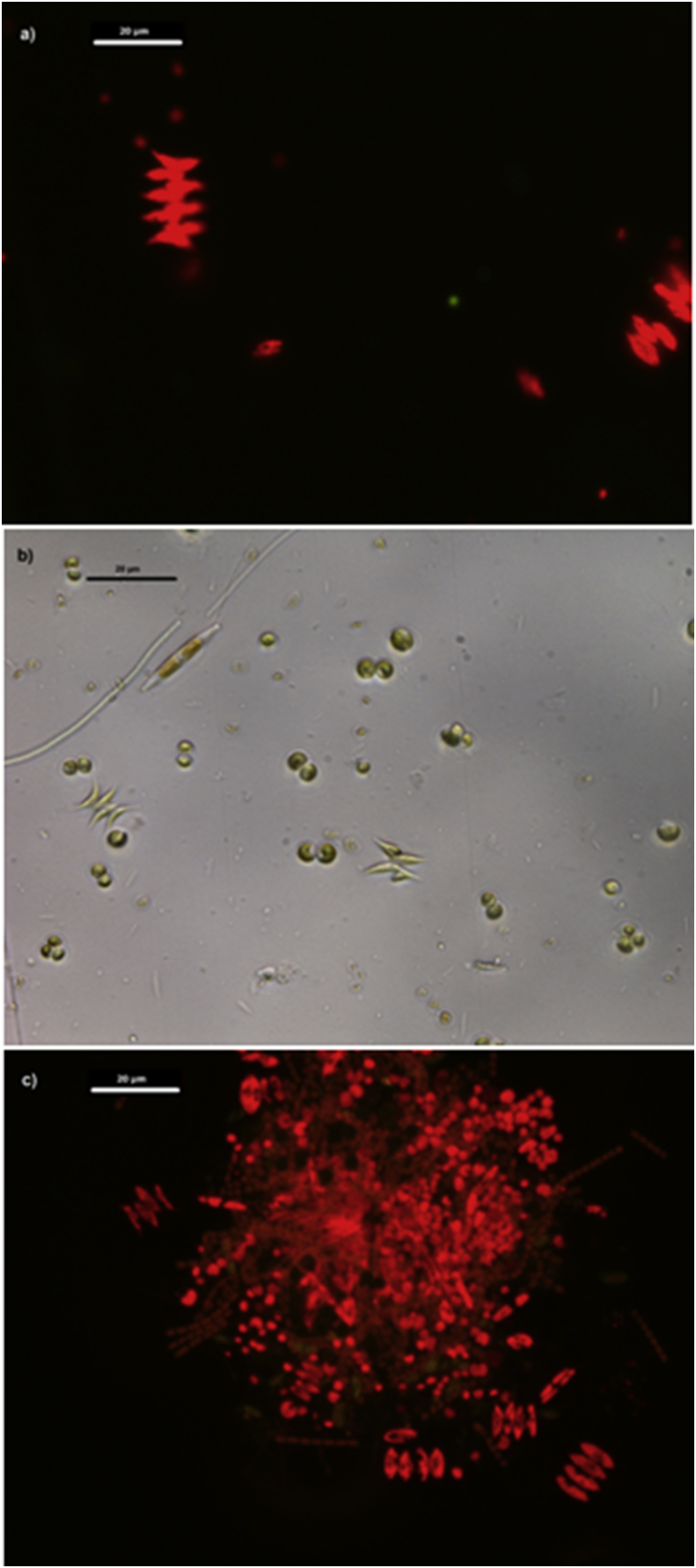
Samples observed under epifluorescence microscope (Leica DM2500/DFC420c digital camera) using a 63× objective. Scale bar = 20 μm. BRT Experiments in González-Camejo et al. [1] a) Experiment BRT4.5: *Scenedesmus* and scarce density of *Chlorella;* b) Experiment BRT6, bright-field image showing *Scenedesmus, Chlorella* and scarce cyanobacteria and diatoms; c) Experiment BRT9, a mixture of *Scenedesmus* and *Chlorella* and high concentration of cyanobacteria.

It must be also considered that operating the MPBR at BRTs out of their optimal range can imply the proliferation of competing organisms (Fig. 4).

## 2 Experimental design, materials and methods

### 2.1 Substrate turbidity

To evaluate the effect of substrate turbidity, lab-scale assays were performed in 500-mL Erlenmeyer flasks, each one of which was lighted at different intensities: 60, 120 and 240 μmol·m^−2^·s^−1^ (measured on the flask surface). These intensities were achieved by varying the number of LED lamps (SevenOn 11w): 1, 2 and 4 lamps, respectively.

The culture for the experiments was composed of 100 mL of microalgae taken from the MPBR plant during the continuous operation of Experiment HRT1, and 100 mL of AnMBR effluent, i.e. microalgae substrate [1]. The initial microalgae biomass concentration was measured by means of optical density of 680 nm (OD680) by a MERC Spectroquant Pharo 300 spectrophotometer, obtaining values of around 1.74–1.80, so that the shadow effect due to microalgae was not considered.

Each assay consisted of several tests in which the turbidity value of AnMBR effluent varied by adding different quantities of kaolin (Table 1). In each test microalgae growth (G) was monitored by measuring the OD680 evolution for two hours. Microalgae growth for each test was calculated as the slope of the line obtained in the OD680 time evolution.

**Table 1:** Substrate turbidity and initial biomass concentration of each test.

	Turbidity (NTU)				
	Test 1	Test 2	Test 3	Test 4	Test 5
240 μmol·m^−2^·s^−1^	0	300	600	1200	3000
120 μmol·m^−2^·s^−1^	0	300	600	1200	3000
60 μmol·m^−2^·s^−1^	0	150	300	1200	3000

### 2.2 Respirometric tests

A 400-mL cylindrical closed PBR was placed inside a climate chamber to carry out the respirometric tests at constant temperature; i.e. 21–23 °C. It was lit by four cool-white LED lamps (T8 LED-Tube 9 w) to supply a light intensity of 100 μmol·m^−2^·s^−1^. An oxygen probe (WTW CellOx 325) monitored the dissolved oxygen (DO) concentration and temperature of the culture during the 30 minutes that each test lasted. Before each respirometric test, bicarbonate (20 mg C·L^−1^) was added to the microalgae sample to avoid carbon limitation. In addition, diluted sulphuric (0.1 M) was injected whenever the pH rose over a set-point of 7.5.

Seven respirometric tests were done at ammonium concentrations of 7, 10, 12, 14, 17, 24 and 27 mg N·L^−1^. Microalgae were obtained from a sample from the MPBR plant during the continuous operation of Experiment BRT4.5 [1]. This sample was diluted with tap water in order to reduce the ammonium concentration up to 7 mg N·L^−1^. To obtain the rest of the ammonium concentrations, the corresponding amount of a standard dilution of 1000 mg NH_4_·L^−1^ was added to microalgae. Similar biomass concentrations; i.e., OD680 in the range of 0.30–0.33 were maintained for all the tests.

OPR was selected as reliable parameter since it has been reported to be proportional to biomass production rate [3].

To calculate the net oxygen production rate (OPR) (mg O_2_·L^−1^·h^−1^), Eq. (1) was used:

(1) $$\frac{dDO}{dt}=k_{L}a\cdot \left(DO_{sat}-DO\right)+OPR$$

where dDO/dt is the variation of oxygen concentration over time (mg O_2_·L^−1^·h^−1^), k_L_a is the oxygen mass transfer coefficient (h^−1^), DO_SAT_ is the oxygen saturation concentration at the culture temperature (mg O_2_·L^−1^), DO is the oxygen concentration in the culture (mg O_2_·L^−1^).

k_L_a was evaluated by doing respirometric tests with clean water as medium in duplicate. An average value of 0.432 h^−1^ was obtained. To calculate the OPR, the minimum square error criterion was used to obtain the optimal fit to Eq. (1) [4].

It must be considered that the OPR obtained by Eq. (1) in a mixed culture like the one used in this study is actually a net value which is composed by several factors: i) microalgae photosynthesis; ii) microalgae respiration; iii) respiration of heterotrophic bacteria; and iv) activity of nitrifying bacteria, both ammonium oxidising bacteria (AOB) and nitrite oxidising bacteria (NOB) [4].

However, activity of heterotrophic bacteria was expected to be low because of the low organic loads of the substrate [1]. Nitrifying bacteria activity was also expected to be low because allylthiourea (ATU) was added to inhibit AOB growth [5]. On the other hand, microalgae respiration was expected to affect all the tests at a similar way because the sample used was the same in all the tests. In conclusion, the net OPR obtained by Eq. (1) was considered as a valid indirect measurement of microalgae activity.

Ammonium concentrations were analysed according to Standard Method 4500-NH3-G [6] in a Smartchem 200 automatic analyser (WestcoScientific Instruments, Westco).

### 2.3 Growth rate under outdoor conditions

Microalgae growth rate (μ) was calculated by applying the Verhulst logistic kinetic model [7] to the OD680 evolution: Eq. (2):

(2) $$\mu =\frac{OD680_{m}\cdot OD680_{0}\cdot e^{\mu \cdot t}}{OD680_{m}-OD680_{0}+OD680_{0}\cdot e^{\mu \cdot t}}$$

where μ is the specific growth rate (d^−1^), OD680_m_, OD680_o_ and OD680 are the optical density of 680 nm at an operation time which corresponded to infinite, zero, and *t*, respectively; and *t* is the time of batch operation (d).

Growth rates were evaluated during the start-up stages of Experiments BRT4.5 and HRT3.5 [1]. In Experiment BRT4.5, the culture was dominated by green microalgae *Scenedesmus* (90% of total eukaryotic cells (TEC)) with low *Chlorella* presence (around 10% of TEC). On the other hand, in Experiment HRT3.5 *Chlorella* was the dominant genus with 90% of TEC, while *Scenedesmus* reached only 10% of TEC. Other microorganisms such as bacteria and cyanobacteria were also present in the inoculums to a lesser extent but were not quantified.
